# Influence of antidepressant use on ^123^I-MIBG heart and lung uptakes in the diagnosis of Lewy body disease

**DOI:** 10.1007/s12149-022-01728-6

**Published:** 2022-02-19

**Authors:** Shinobu Adaniya, Miwako Takahashi, Keitaro Koyama, Kenichiro Ogane, Toshimitsu Momose

**Affiliations:** 1 Department of Nuclear Medicine, Graduate School of Medicine, The International University of Health and Welfare, Narita, Japan; 2 Department of Advanced Nuclear Medicine Sciences, Institute for Quantum Medical Science, National Institutes for Quantum and Radiological Science and Technology, 4-9-1 Anagawa, Inage-ku, Chiba 263-8555 Japan

**Keywords:** Lewy body disease, MIBG, Lung, Antidepressant, Serotonin transporter

## Abstract

**Objective:**

The clinical significance of decreased physiological lung uptake of ^123^I-metaiodobenzylguanidine (MIBG) has not been well investigated. This study aimed to elucidate the association between a decrease in lung MIBG uptake with antidepressant intake and the myocardial MIBG uptake in patients who were clinically diagnosed with Lewy body disease (LBD) and patients who were diagnosed as not having LBD.

**Methods:**

We retrospectively reviewed the heart and lung uptakes on 167 consecutive MIBG scans, antidepressant status, and clinical diagnosis of LBD. The images were visually classified into two groups: decreased lung uptake and preserved lung uptake. A semi-quantitative analysis was performed using the heart-to-mediastinum ratio (H/M), lung-to-mediastinum ratio (L/M), and myocardial washout rate (WR).

**Results:**

All 17 patients with decreased lung uptake were on treated with antidepressants, while none of the 150 patients with preserved lung uptake were treated with any antidepressants. Of the 17 patients with decreased lung uptake, 6 patients were clinically diagnosed as LBD and other 11 were clinically diagnosed as non-LBD. There was not significant difference in early H/M, delayed H/M, and myocardial WR between the 11 non-LBD patients with decreased lung uptake and 83 non-LBD patients with preserved lung uptake (2.87 ± 0.69 vs. 2.89 ± 0.44, 3.09 ± 0.48 vs. 2.98 ± 0.59, and 21.8 ± 11.3% vs. 21.1 ± 12.5%, respectively). Moreover, in LBD patients, there were no significant differences in those values between six patients with decreased lung uptake and 67 patients with preserved lung uptake (1.68 ± 0.32 vs. 1.73 ± 0.42, 1.34 ± 0.21 vs. 1.54 ± 0.57, 46.2 ± 22.8% vs. 42.8 ± 21.3%, respectively).

**Conclusions:**

Antidepressants probably blocked MIBG uptake in the lungs, and a decreased lung uptake was not significantly associated with heart uptake. A remarkable decrease in lung uptake can be a signal to check a patient’s medication status.

## Introduction

Scintigraphy using ^123^I-metaiodobenzylguanidine (MIBG), a norepinephrine (NE) analog, is helpful for discriminating Lewy body disease (LBD) from other neurodegenerative disorders [1–3]. MIBG is taken up by the sympathetic nerve terminals through catecholamine transporters but is not metabolized; therefore, it accumulates in the sympathetic nerve terminals. Myocardial MIBG accumulation can reflect sympathetic nervous integrity, and MIBG scintigraphy is currently among the indicative biomarkers of dementia with Lewy body diagnostic criteria [4].

Myocardial MIBG accumulation is decreased in patients with LBD because Lewy body pathology involves the peripheral autonomic nervous system as well as the central nervous system [5, 6]. Although the lung also shows physiological MIBG uptake, it is not related to LBD [7–9]. We previously reported two cases in which MIBG lung uptake remarkably decreased with the use of a selective serotonin reuptake inhibitor (SSRI)/serotonin noradrenaline reuptake inhibitor (SNRI) and was preserved during the medication-naive or withdrawal state [10]. These findings suggest that MIBG uptake in the lungs may be affected by antidepressants through serotonin transporters (SERTs) and norepinephrine transporters (NETs). In addition, elucidating whether the decrease in MIBG lung uptake is associated with the decrease in cardiac accumulation is important in the diagnosis of LBD.

In this study, we retrospectively investigated MIBG scans to investigate the lung MIBG uptake in relation to antidepressant intake and its effect on the heart-to-mediastinum ratio (H/M), which is commonly used to assess cardiac MIBG accumulation.

## Materials and methods

We identified 169 consecutive MIBG scans performed as part of the clinical evaluations for the diagnosis of LBD at our hospital between April 2016 and March 2021. A clinical diagnosis of LBD was established based on the Movement Disorder Society clinical diagnostic criteria for PD [11] and the DLB Consortium Consensus criteria [4]. The exclusion criteria were as follows: patients treated for cardiac disease, pulmonary disease, or severe diabetes mellitus because they also affect MIBG uptake in the heart and lungs; and patients on reserpine or labetalol, which are known to interact with MIBG heart uptake, and images with no motion artifacts. Consequently, two scans among those conducted, one with a motion artifact and the other with severe emphysema, were excluded. Thus, 167 total scans were included in this study. Of them, scans were performed twice for five patients and three times for one patient. Therefore, while the number of patients and the number of scans differed, we considered each scan as related to an individual patient. This study was performed in accordance with the Declaration of Helsinki and was approved by the institutional review board of the institute’s hospital. Informed consent was obtained from all patients.

### Imaging protocol

A dose of 111 MBq (3 mCi) of ^123^I-MIBG (MyoMIBG^Ⓡ^-I123 Injection; FUJIFILM Toyama Chemical Co., Ltd., Chuo-ku, Tokyo) was injected intravenously. With the patient in the supine position, anterior chest planar images were obtained 15 min (early image) and 3 h (delayed image) after the injection. Until October 2018, the scans were performed using a dual-head gamma camera (Millennium MG; GE Healthcare, Milwaukee, WI, USA) equipped with a low-energy general-purpose (LEGP) collimator with a 360 mm × 510 mm field of view (256 × 256 matrix size), 9.7% energy resolution, and 3.9-mm spatial resolution full-width at half-maximum (FWHM). After October 2018, a dual-head gamma camera (Discovery NM630; GE Healthcare) equipped with a medium-energy general-purpose (MEGP) parallel-hole collimator with a 400 mm × 500 mm field of view (128 × 128 matrix size), 9.7% energy resolution, and 3.9-mm spatial resolution FWHM was used. All scans were performed for 3 min with an energy window of 159 keV ± 10%.

### Visual evaluation

Two nuclear medicine experts visually evaluated the MIBG images and classified them into two groups: decreased lung uptake or preserved lung uptake. When the lung MIBG uptake was almost the same as the background, it was judged as decreased.

### Semi-quantitative evaluation

**Fig. 1:**
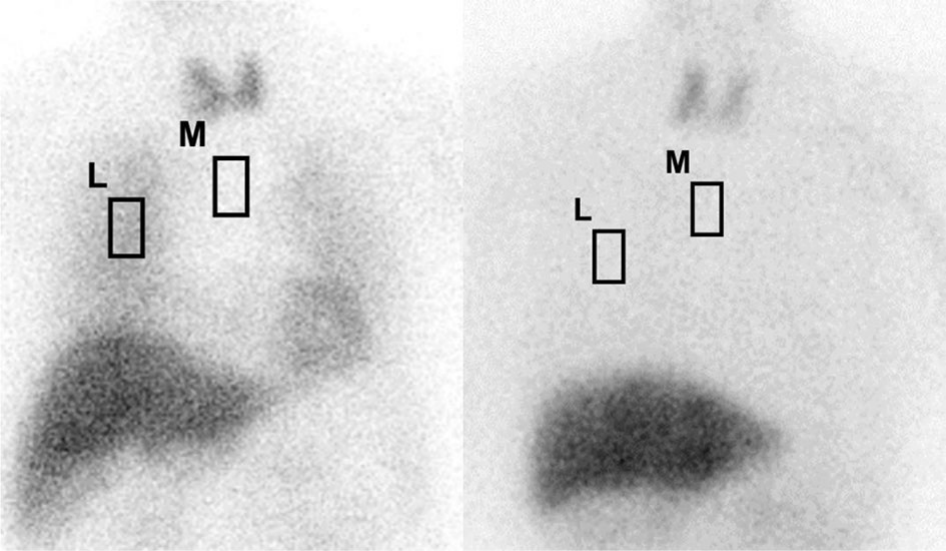
Manual ROI placement on the right lung and mediastinum in a patient with normal MIBG uptake (left) and with decreased uptake in the lungs and heart (right). The ROI size is a 3 × 5 cm square. L, lung. M, mediastinum

We calculated the H/M and the lung-to-mediastinum ratio (L/M) as semi-quantitative values of heart and lung MIBG uptake, respectively. Regions of interest (ROIs) were placed on the heart and upper mediastinum area using the software Smart MIBG (FUJIFILM Toyama Chemical Co., Ltd.), in which the H/M values were standardized between the two different gamma cameras. The conversion coefficient was 0.64 for the former gamma camera with the LEGP collimator, and 0.90 for the current gamma camera with the MEGP collimator, as obtained via dedicated phantom experiments [12]. We also placed 3 × 5 cm square ROIs manually on the right middle lung field to avoid superimposition with heart accumulation and the mediastinum (Fig. 1). Since the digital data available for the current computational system had not been stored, manual ROI placement could not be performed for the data obtained prior to October 2018, the L/M was calculated only for the 70 scans obtained after the camera was changed.

We calculated the myocardial washout rate (WR) as follows:

$$ {\text{WR}} = \frac{{({\text{He}} - {\text{Me}}) - ({\text{Hd}} - {\text{Md}})}}{{{\text{He}} - {\text{Me}}}} \times 100 \left[ \% \right], $$

where *He* and *Hd* were the mean counts of the heart ROIs in the early and delayed images for each patient, respectively. Similarly, *Me* and *Md* were the mean counts of the mediastinum ROIs in the early and delayed images, respectively. For the calculations, these counts were corrected for decay from the time of the injection. When the lung and heart uptakes were very low, the ROIs were carefully placed on the lung and heart by visually comparing the chest X-p of each patient. The mediastinum ROI for H/M calculation was automatically generated using Smart MIBG, but was adjusted manually when the position was located outside of the mediastinum.

### Statistical analysis

L/M was compared between the decreased lung uptake and preserved lung uptake groups. Next, in the patients who were not diagnosed with LBD (non-LBD), H/M and myocardial WR were compared between the decreased and preserved lung uptake groups; the same comparison was performed in the patients with LBD. These numerical comparisons were performed using the t-test or Mann–Whitney *U* test when the distribution was non-parametric. Statistical significance was set at a two-tailed *p* < 0.05. All statistical analyses were performed using the Statistical Package for the Social Sciences (SPSS) software version 26 (IBM Corporation, Armonk, NY, USA).

## Results

**Fig. 2:**
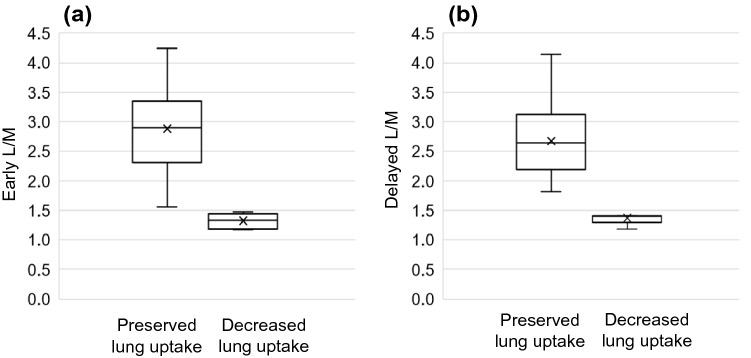
Box-and-whisker plot of the early lung-to-mediastinum ratio (L/M) (**a**), delayed L/M (**b**) for 70 MIBG scans according to the visual judgment on the lung MIBG uptake. The values of L/M values are significantly decreased in the group judged as having visually decreased lung uptake (*t*-test; *p* = 0.007 and 0.011, respectively). X mark; average value, central horizontal line; median value, horizontal ends of the box; upper and lower quartiles, the bar end; maximum and minimum values

All 167 MIBG images were visually classified into two groups based on MIBG lung uptake: 150 with preserved lung uptake and 17 with decreased lung uptake. On all of the images, two nuclear medicine experts’ judgments were agreed upon. L/M were available for 70 patients, including 64 preserved lung uptake scans and 6 decreased lung uptake scans; early L/M and delayed L/M were significantly lower in the decreased lung uptake scans than in the preserved lung uptake scans (early L/M and delayed L/M, 1.32 ± 0.14 and 1.37 ± 0.14 vs 2.88 ± 0.71 and 2.67 ± 0.57, respectively), a finding that is consistent with the visual classification. A box-and-whisker plot of the L/M is shown in Fig. 2.

While none of the 150 patients with preserved lung uptake was treated with any antidepressants, all 17 patients showed a decreased lung uptake with antidepressant use, of whom 11 were taking an SNRI (duloxetine; 20–40 mg/day), 4 were taking an SSRI (sertraline 100 mg/day, escitalopram 10 mg/day, paroxetine 10 mg/day, fluvoxamine; 150 mg/day, each), and 2 were taking a tricyclic antidepressant (TCA) (clomipramine 20 mg/day). All these patients were treated with these drugs for more than 1 month; thus, we considered that they underwent MIBG scan under the steady-state plasma concentrations of each antidepressant. One patient who underwent MIBG scintigraphy 5 days after discontinuing an SNRI (duloxetine 20 mg/day) was classified into the non-medication category because the 5-day period was considered sufficient for drug withdrawal from the body based on the drug’s 10-h half-life.

**Fig. 3:**
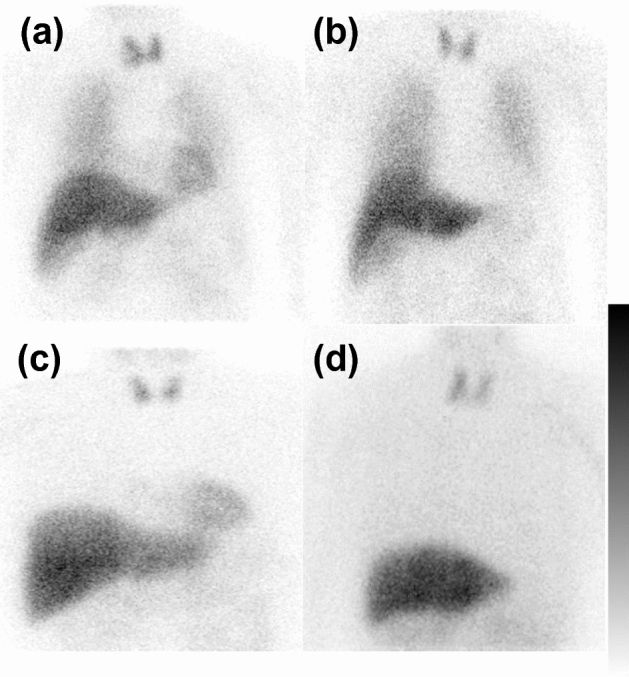
Representative images of preserved lung uptake in patients who were diagnosed as not having Lewy body disease (non-LBD) (**a**), preserved lung uptake in patients with Lewy body disease (LBD) associated with decreased heart uptake (**b**), decreased lung uptake in patients with non-LBD (**c**), and decreased lung uptake in patients with LBD associated with decreased heart uptake (**d**)

**Fig. 4:**
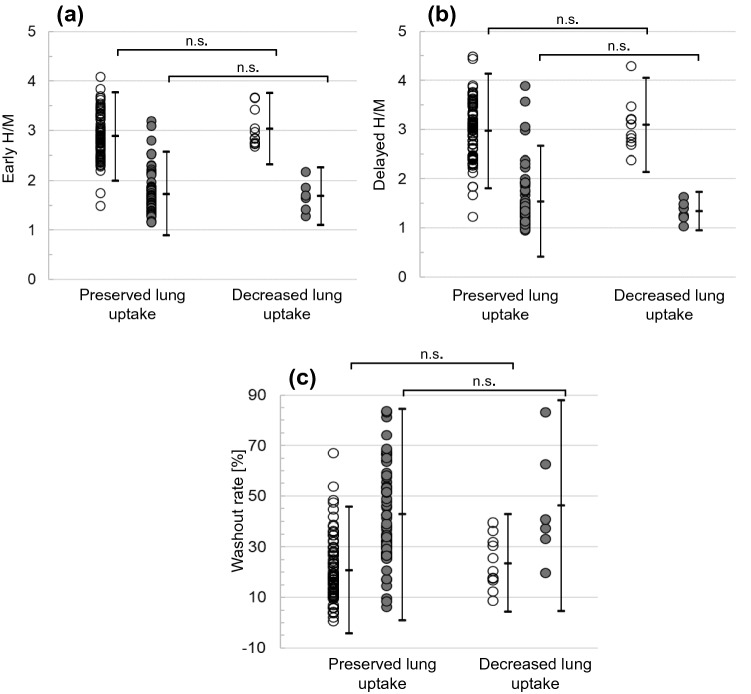
Early heart-to-mediastinum ratio (H/M) (**a**), delayed H/M (**b**), and myocardial washout rate (**c**) are plotted according to the preserved lung uptake and decreased lung uptake groups, with the bars representing the average ± 2 SD. White circle: patients who were diagnosed as not having Lewy body disease; gray circle: patients with Lewy body disease; n.s.: not significant

The distribution of patients according to the clinical diagnoses, antidepressant medication status, early H/M, delayed H/M, and myocardial WR are summarized in Table 1. Representative images of preserved lung uptake patients with non-LBD and those with LBD as well as decreased lung uptake in patients with non-LBD and those with LBD are shown in Fig. 3. The plots of early and delayed H/M and myocardial WR in individual patients are shown in Fig. 4. There were no significant differences in early H/M, delayed H/M, and myocardial WR between patients with preserved versus decreased lung uptake among the non-LBD patients (p = 0.22, 0.47, 0.44, respectively) as well as no significant differences in these values between patients with preserved versus decreased lung uptake among the LBD patients (p = 0.75, 0.10, and 0.74, respectively).

**Table 1:** A summary of patient numbers based on ^123^I-metaiodobenzylguanidine (MIBG) lung uptake, clinical diagnosis, and antidepressant status

		Medication + (*n*)	Age (years)	Male:female	Early H/M	Delayed H/M	Myocardial washout rate (%)
Preserved lung uptake (*n* = 150)	Non-LBD (*n* = 83)	0	72.0 ± 12.0	41:42	2.89 ± 0.44	2.98 ± 0.59	21.1 ± 12.5
	LBD (*n* = 67)	0	72.3 ± 10.1	36:31	1.73 ± 0.42	1.54 ± 0.57	42.8 ± 21.3
Decreased lung uptake (*n* = 17)	Non-LBD (*n* = 11)	11	73.8 ± 11.8	3:8	2.87 ± 0.69	3.09 ± 0.48	21.8 ± 11.3
	LBD (*n* = 6)	6	72.5 ± 14.6	2:4	1.68 ± 0.32	1.34 ± 0.21	46.2 ± 22.8

*H/M* heart-to-mediastinum ratio, Medication +: Patients were being treated with antidepressants when ^123^I-MIBG scintigraphy was performed

## Discussion

In this study, 167 MIBG scans were retrospectively investigated to assess MIBG lung and heart uptake in relation to LBD and antidepressant use. Our findings showed that the use of antidepressants, including SSRIs, SNRIs, and TCAs, was associated with decreased MIBG lung uptake; in patients with non-LBD, while the lung uptake was decreased, the heart uptake was preserved. It was assumed that antidepressant intake may not interfere with the diagnostic value of MIBG scintigraphy at the doses used in our patients.

MIBG physiological uptake is observed in the lungs as well as the heart. However, unlike myocardial MIBG uptake, lung MIBG uptake has not been related to the pathological status of LBD [7–9]. In this study, lung uptake was decreased in all the patients who were treated with SSRIs, SNRIs, and TCAs, suggesting that MIBG uptake in the lung is involved with catecholamine uptake sites including SERTs. Blom et al. demonstrated that MIBG accumulation in human platelets, which express SERTs, was reduced by SSRI, suggesting that MIBG has an affinity for SERTs [13]. Therefore, SSRIs may interfere with MIBG lung uptake at the SERT sites. In a human study of the use of ^11^C-cyanoimipramine—an imipramine derivative with potent SERT inhibitor activity—positron emission tomography images showed the highest uptake in the lung, suggesting that SERT expression is abundant in the human lung [14].

Serotonin is a vasoconstrictor that acts on the peripheral vascular muscles [15]. The ability of the lung to remove serotonin from the pulmonary circulation underlines the importance of the lung in preventing vasoactive substances from accessing the systemic circulation. The process of this removal was revealed by experiments using ex vivo rat lungs with specific drugs such as cocaine, imipramine, and chlorpromazine to inhibit pulmonary uptake of radiolabeled serotonin [16], where the uptake site was localized mainly on the endothelium as evidenced on electromicroscopy with radiolabeled serotonin [17, 18]. A previous study estimated the extraction rate of serotonin from the pulmonary circulation to the left atrium of heart in patients undergoing pulmonary cardiovascular bypass as 65%, which was higher than that of NE (23%) [19]. In a rodent study, MIBG pulmonary extraction was reduced following endothelial injury [20]. These studies suggest the existence of SERTs in pulmonary endothelial cells that remove serotonin from the pulmonary circulation.

When considering the reason why the lung uptake was remarkably decreased while the heart uptake was preserved in patients taking antidepressants in this study, we paid attention to the dose of the antidepressants, which were relatively low. Catecholamine uptake systems are classified into two types [21, 22]: one is a sodium-dependent system, which is characterized as saturable with high affinity and low capacity, and the other is a sodium-independent system, which is characterized as unsaturable and energy-independent. Tobes et al. investigated the relationship between the percent inhibition of these uptake systems by tricyclic antidepressants and the dose of tricyclic antidepressants, showing that the percent inhibition of the sodium-dependent system reached almost 100% at a low concentration of tricyclic antidepressants, while the percent inhibition of the sodium-independent system was below 10% at the same dose of tricyclic antidepressants [22]. Therefore, we believe that the differences in MIBG uptake in the lung and heart associated with antidepressant intake may be explained by the predominance of these two catecholamine uptake systems. Our results suggest that MIBG uptake in the lung is predominantly processed by a sodium-dependent system with saturable and low-capacity characteristics.

**Fig. 5:**
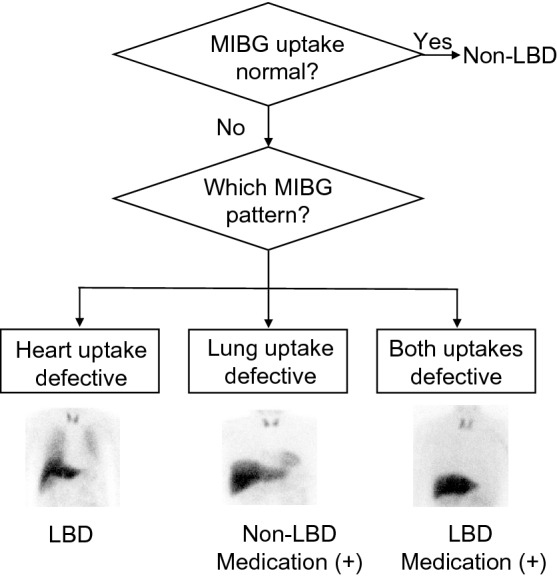
A diagram of the interpretation based on ^123^I-metaiodobenzylguanidine lung and heart uptake patterns. The decreased lung uptake and both decreased patterns suggested that the patient was taking a serotonin reuptake inhibitor, serotonin noradrenaline reuptake inhibitor, or tricyclic antidepressant

We found four patterns of MIBG uptake in this study: normal, decreased heart uptake (but preserved lung uptake), decreased lung uptake (but preserved heart uptake), and both uptakes decreased. This classification can be helpful in diagnosing LBD in patients while considering their medication status because all patients with decreased lung uptake in this study were taking antidepressants. A possible diagram is shown in Fig. 5. For example, a decreased lung uptake pattern may indicate that the patient is taking an SNRI, SSRI, or TCA, and we should consider the possibility of a patient suffering from drug-induced parkinsonism. The medication doses of patients exhibiting both decreased patterns must also be assessed because relatively high SNRI doses may also affect the accumulated uptake in the heart [23]. Antidepressants were recently used for the treatment of lower back pain and depression [24]. In this study, one patient was treated with an antidepressant for back pain. This suggests that when assessing the medication status of antidepressants, we should consider the possibility that the antidepressants were prescribed by orthopedics for back pain, not depression. We believe that our findings will be helpful for diagnosing LBD in patients treated with antidepressants.

The limitations of our study were that only a small number of patients were taking antidepressants and that the doses of these agents were relatively low. Although our results suggested that antidepressant therapy did not affect heart MIBG uptake, a generalized conclusion can only be reached after further research is conducted on patients treated with higher doses of antidepressants. Furthermore, to confirm the effect of antidepressant intake on MIBG scintigraphy, it is necessary to compare between MIBG scans with and those without antidepressant use in the same patient. This study included two cases of such patients as we reported previously [10]. Briefly, one patient maintained cardiac uptake on the first MIBG scintigraphy, but three years later, her symptoms proceeded and required further examination. Since antidepressant treatment for her symptoms before the second MIBG scintigraphy was inevitable, the second MIBG scan showed decreased lung uptake while preserved heart uptake. The other patient showed decreased heart uptake on the first MIBG scintigraphy while taking antidepressants. At that time, the effect of the antidepressant was not denied; therefore, after discontinuing the antidepressant for 5 days, MIBG scintigraphy was repeated and showed decreased heart uptake but preserved lung uptake. Of course, we should have conducted MIBG scintigraphy after the discontinuation of antidepressants in more patients, but such discontinuation exacerbated the patient’s symptoms, and performing two MIBG examinations would increase the radiation exposure. Therefore, we did not conduct such interventions in this study.

In conclusion, our results suggest that MIBG uptake can be blocked by antidepressants in the lungs but not significantly in the heart. Making a diagnosis using MIBG scintigraphy for LBD as an indicative biomarker is possible in patients who are taking antidepressants, but the effect of relatively high doses of antidepressants remains to be investigated. MIBG lung uptake can act as a signal to check the patient’s medication status.
